# The Maxillary Sinus Membrane Elevation Procedure: Augmentation of Bone around Dental Implants without Grafts—A Review of a Surgical Technique

**DOI:** 10.1155/2012/105483

**Published:** 2012-06-18

**Authors:** Christopher Riben, Andreas Thor

**Affiliations:** Department of Plastic & Oral and Maxillofacial Surgery, Institute of Surgical Sciences, Uppsala University, 751 85 Uppsala, Sweden

## Abstract

*Background*. Long-term edentulism may in many cases result in resorption of the alveolar process. The sinus lift procedure aims to create increased bone volume in the maxillary sinus in order to enable installation of dental implants in the region. The method is over 30 years old, and initially autogenous bone grafts were used and later also different bone substitutes. Since 1997, a limited number of studies have explored the possibility of a graftless procedure where the void under the sinus membrane is filled with a blood clot that enables bone formation. *Aim*. To describe the evolution of the sinus-lift technique and to review the literature related to the technique with a focus on long-term studies related to the graft-less technique. *Methods*. The electronic database PubMed was searched, and a systematic review was conducted regarding relevant articles. *Results*. A relatively few long-term studies using the described technique were found. However, the technique was described as reliable considering the outcome of the existing studies. *Conclusion*. All investigated studies show high implant survival rates for the graftless technique. The technique is considered to be cost-effective, less time-consuming, and related to lower morbidity since no bone harvesting is needed.

## 1. Introduction

The aim of this paper is to describe the evolution of the sinus membrane elevation technique, from when the concept of sinus lift was first reported in the literature in 1976, to the present, where bone can be formed around implants placed in the sinus floor using only blood and no other augmentation material. The paper describes the proposed mechanisms that make this technique possible. Since the technique can be less time-consuming regarding periods and involves less morbidity for the patient, it is of interest to review the scientific data of this specific procedure.

## 2. Strategy of Literature Search on the Graftless Sinus Augmentation (Sinus Membrane Elevation Technique)

The PubMed database was searched to locate studies related to sinus lift surgery in general and sinus lift without the use of graft in specific. Articles were searched from 1997 (the first known study of sinus lift surgery without the use of grafts [1]) to November 2011.

### 2.1. PubMed Search

The free test words *sinus *and* implant *were used. References in relevant publications were also examined. There were no language restrictions.

### 2.2. Search in Reference List

The reference list of all included articles was searched for relevant clinical trials.

### 2.3. Inclusion and Exclusion Criteria

Clinical human studies with a minimum followup of one year or more were included. Retrospective and prospective studies were included. Studies were excluded when bone graft or bone substitutes had been used in relation to sinus lift surgery or when the osteotome technique was used. Due to the low number of relevant articles, lack of randomization or control group did not pose a reason for exclusion.

## 3. Methods and Review

### 3.1. Study Selection

The authors (CR and AT) who together performed the study selection were not blinded to the publishing journal, the authors, or the institution. Titles and abstracts were assessed to determine whether an article was meeting the predetermined criteria. When this was not enough to make a decision, the full article was retrieved and examined and a decision on inclusion in the paper was finalized.

Data collection from the included studies was done without blinding to the publishing journal, the authors, or the institution.

Articles investigating and discussing sinus lift surgery, however, not directly related to the described sinus elevation technique, found during the literature search or previously known to the authors, were included for the general overview.

### 3.2. Background on Sinus Lift

During long-term edentulism, resorption of the alveolar process occurs. Since the maxillary sinus also pneumatises during these circumstances [2], the remaining bone volume can become very small and therefore clinicians and researchers have continuously developed techniques to overcome this problem.

The sinus lift is a surgical procedure aiming to create an increased bone volume in the maxillary sinus floor in order to enable installation of fixtures in the region. The graft in the sinus bottom may be left to heal primarily before implants are placed in a second surgery (2-stage procedure), or implants may be placed simultaneously with the graft (1-stage procedure). The grafts are, however, exposed to a rather substantial degree of resorption [3].

The technique of sinus lift was first orally reported in 1976 by Tatum [4] and first published in 1980 by Boyne and James [5] and subsequently also by Tatum [6]. The surgical procedure has undergone development, and variations exist. Autogenous bone, regarded as the preferred option but with an important drawback of an unpredictable rate of resorption, has later been replaced by many surgeons by the use of bone substitutes [7]. The range of different materials installed and explored in the sinus is impressive. Later works have included trials of rhBMP-2 [8] as well as the use of mesenchymal stem cells (MSCs) in combination with inorganic bovine bone [9]. Long-term followup showing satisfying results regarding implant survival using two commonly used techniques, the lateral sinus floor elevation technique and the osteotome technique, was presented in 2010 by Tetsch et al. They followed 983 patients with 2190 implants over a time period of 176 months using Kaplan-Meier analysis and showed an implant survival rate of 97,1% [10].

### 3.3. Surgical Technique

The basic surgical principle and technique have not significantly changed. Intraoral access to the maxillary sinus is gained through the oral mucosa in the region of the anterior maxillary sinus wall. A bony window is prepared, and the sinus membrane is dissected and lifted from the sinus floor in order to enable insertion of a graft alone, or around installed implants to facilitate bone formation in the created secluded space. The bony window has mostly been kept attached to the membrane and elevated superiorly.

The sinus lift surgical technique has developed over time, and several minor variations now exist. The surgery is commonly performed under local anaesthesia and sedation.

### 3.4. Sinus Lift Surgery with Simultaneous Installation of Implants without the Use of Grafts

**Figure 1:**
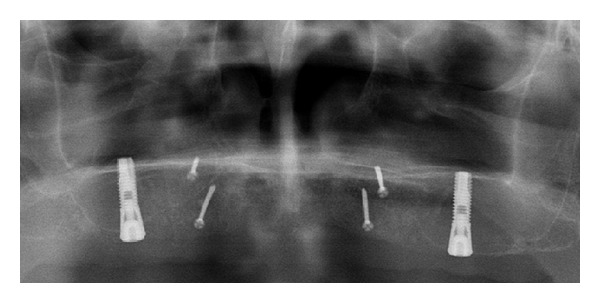
One-week postoperative baseline panoramic view over reconstructed atrophic maxilla. Block bone grafts attached with titanium screws in the anterior and sinus membrane elevation performed in the maxillary sinus floor. Notice the minute amount of bone (1-2 mm) in the sinus floor. The conical shape of the marginal part of the implant represents 5 mm.

**Figure 2:**
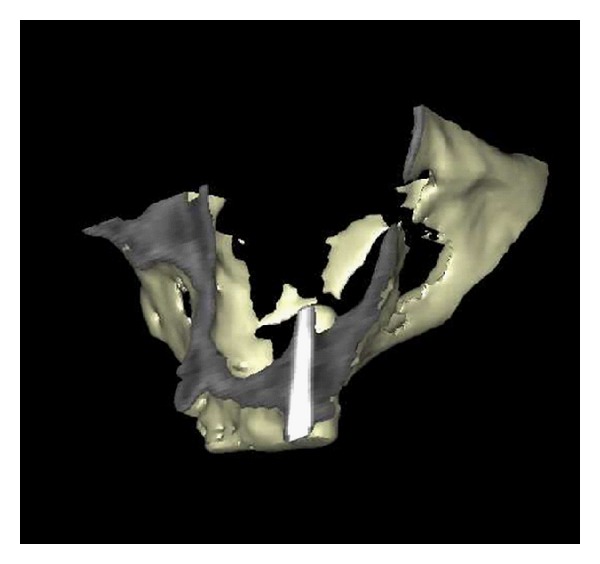
3 D reconstruction of CT scan from the same patient as in Figure 1 six months postoperatively, left side. Bone is formed around implants in the maxillary sinus floor.

**Figure 3:**
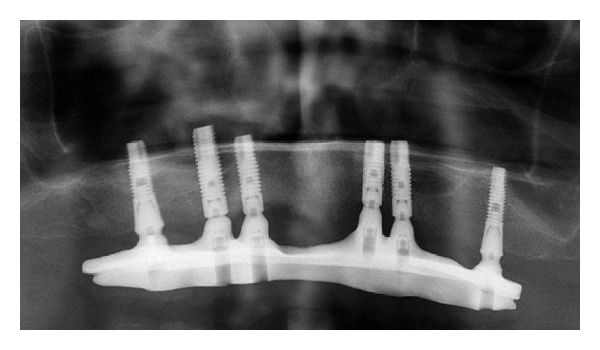
Situation 3 years postoperatively.

**Figure 4:**
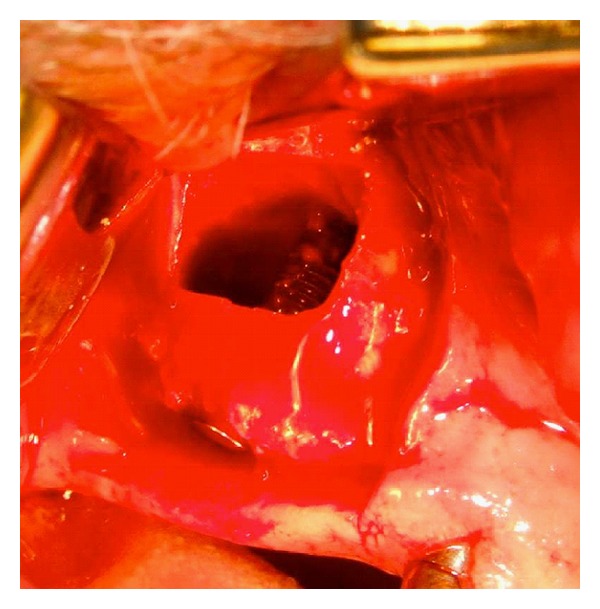
Surgical technique. An osteotomy is performed, and the bony window is temporarily removed. The installed implant is here seen elevating the sinus membrane, and, after blood has filled the created compartment around the implant, the bony window is thereafter replaced.

For over 30 years, extensive experimental and clinical research has been undertaken based on the idea of necessity of grafting the maxillary sinus and great industrial investments have been made into developing products for this area [11]. Eventually, the idea of a graftless augmentation of the maxillary sinus has evolved (Figures 1, 2, 3, and 4).

Boyne presented experimental results from a primate study in 1993 in which implants were left without grafts to protrude 5 mm into the sinus floor and experienced bone formation [12].

In 1997, Ellegaard and colleagues described a technique whereby 80 fixtures were installed in the posterior maxilla in 24 periodontally compromised patients, of which 38 involved surgery of the maxillary sinus [1]. A circular fenestration was prepared in the lateral antral wall, at least 5 mm superiorly to the estimated maxillary sinus floor. Thereafter, the sinus membrane was dissected around the fenestration as well as from the floor of the maxillary sinus. The implants were otherwise conventionally installed through the remaining alveolar crest. The sinus membrane was left resting on the installed protruding implants, creating a secluded void filled with blood, forming around and between the implants. The repositioned flap covered the prepared window in the antral wall, and no barrier membrane was placed over the bony defect created for entrance to the sinus. In the study, a note was made of the newly formed bone seen around the upper part of the implants protruding up into the sinus cavity on follow-up radiographs. After 5-6 months of healing, the implants were functionally loaded. Of the 38 implants in the maxillary sinus, 35 were successfully integrated during the follow-up time of 27 months.

In 2001, a report at the yearly convention of the Swedish Dental Association by Lundgren included reference to a patient who was planned to initially have a mucosal cyst of the maxillary sinus removed with subsequent augmentation of the maxillary sinus to facilitate implant placement [13]. The cyst was removed through a prepared bony window in the lateral antral wall, and the ruptured mucosa was sutured. The bony window was then replaced and a space secluded by bony walls, and sinus membrane had been created. After 3 months of healing, clear signs of bone formation were observed.

Inspired by this outcome, Lundgren et al. presented a study in 2004 where 19 implants were installed in 12 maxillary sinuses [14]. The bony window was dissected from the underlying sinus membrane and placed in sterile saline solution. The sinus membrane was then dissected from the floor of the maxillary sinus to create the secluded space for the implants. The implants were installed, the bony window was replaced, and the flap was sutured into position. During the follow-up period and at the final evaluation after 12 months, all implants showed stability and bone formation in the maxillary sinus. 

Ellegaard et al. presented a followup of the study from 1997 [1] in which all patients treated during 1990–2002 were examined [15]. Of 262 implants, 131(50%) had been placed in the maxillary sinus. The conclusion of the study was that implants in periodontally compromised patients could be installed in the maxillary sinus with success rate similar to that of conventional implants over a long follow-up period. In a study by Thor from 2007, 20 patients who had received 44 Astra Tech implants in the maxillary sinus were followed annually for up to four years (mean 27.5 months and range 14–45 months) [17]. A sinus lift procedure was considered when the subantral bone was 5 mm or less (mean residual bone height 4.6 mm, range 2.0–9.0 mm). The survival rate of implants evaluated after an average time of 27.5 months was 97.7%. The average amount of bone formation in the maxillary sinus was 6.5 mm. It was concluded that greater bone formation was related to longer implants installed and lower preoperative bone height in the subantral region.

Chen et al. published a study in 2007 of 47 implants in 33 patients evaluated after 2 years [16]. Unlike Ellegaard et al. who removed bone tissue in the region to gain access to the maxillary sinus, the sinus mucosal membrane was here elevated with the bony window still attached to the membrane (folded up into the sinus) and the implants served as tentpoles and space holders. No graft except blood was used, and preoperative bone of 7.5 ± 2.1 mm was reported (measured on panoramic X-ray). After 6 months of healing there were no failures and the average bone gain was 4.5 mm.

Hatano et al. presented a case series of 6 patients in whom successful new bone formation was found in all sinuses after a healing period of 6 months for the implants and an observation period of up to 34 months [18]. In addition, blood clot formation in the compartments around the implants was secured via an injection of peripheral blood and medical glue for closure of the potential gap in the bony window of the osteotomy. In a study by Sohn et al. from 2008, 21 implants inserted in 10 patients were evaluated after 6 months [19]. All implants remained stable during the study period, and bone formation was found in both radiographic and histologic evaluations.

Balleri et al. presented a study where 28 Astra Tech implants had been evaluated after one year [20]. The average baseline bone level was 6.2 mm. No implants were, lost and the average bone gain was 5.5 mm. It was concluded that the bone gain was less than the average lift of the membrane lift (8.2 mm) and that the length of the implants was not related to the amount of gained bone. Also, the bone regeneration was less at the distal aspect of the most posterior-placed implant which could be explained by the theory that this surface was more exposed for the pneumatisation of the sinus.

Jensen and Terheyden recently reviewed bone augmentation techniques related to implant treatment as described by the 4:th ITI Consensus Conference from 2009 [23] and identified 179 sinus augmentation studies using the lateral window technique. Of the 47 studies that fulfilled the inclusion criteria, only three presented data on the graftless technique considered in this paper [14, 16, 17]. All three studies report survival rates (evaluation period of 12–27.5 months) within the range of 97.7% to 100% (110 implants in 63 patients). In their concluding remarks and despite the low number of extant studies, Jensen and Terheyden considered this technique to be a well-documented procedure for maxillary sinus floor elevation.

Recently in 2011, Lin et al. presented a study where 44 patients with 80 implants in the maxillary sinus were followed for five years after delivery of the prosthesis [21]. The survival rate was 100% after five years. The average residual bone height was 5.1 mm before treatment and at least 3 mm was required for inclusion. The average gained bone height after five years was 7.4 mm in the sinus. Also, in 2011, Cricchio et al. presented a study where 189 implants had been installed in the maxillary sinus in 84 patients [22]. A two-stage technique was used in the majority of the cases, 78. The range of the followup was 1–6 years. The survival rate was 98,7%, and the average new bone formation was 5.3 mm after 6 months of healing. Resonance Frequency Analyses showed adequate primary stability and small changes over time.

A summary of studies of sinus lift with blood only is presented in Table 1.

### 3.5. Studies Where the Reported Bone Height Is Low under the Maxillary Sinus

Lundgren et al. [14] reported results from patients with mean bone levels of 7 mm (range 4–10 mm) and the use of Brånemark type implants (19 implants, *Ø* 3.75 mm, TiUnite, Nobel Biocare, Gothenburg, Sweden). Ellegaard et al. reported on patients with as little as 3 mm [1], and Sohn et al. reported on cases in their paper where pretreatment bone levels varied from 1–9 mm [19]. The paper by Chen et al. included patients with at least 5 mm (mean of 7.5 mm ± 2.1 mm). Primary stability was easily achieved in the remaining bone [16]. Hatano et al. reported on 6 patients in whom the thickness of the basal bone ranged from 2 to 10 mm preoperatively, and Brånemark type implants were used in a standard implant drilling protocol [24]. 

Thor et al. [17] included patients with a minimum of 2 mm of remaining bone. The implant installation protocol was therefore altered to achieve primary stability. By using a conical implant with microthreads over the superior 5 mm, sufficient primary stability was achieved in the remaining bone (44 implants, *Ø* 4.5 mm and 5.0 mm, Fixture Microthread ST, Astra Tech AB, Mölndal, Sweden). In order to optimize the primary stability of the implant, a “press-fit” effect was achieved due to a modified drilling protocol. The implant site was thus less widened with the burr than the standard recommended size of the site. The implant was allowed to engage enough in even minimal amounts of remaining subantral bone. The length of the implant was also important as a longer implant may be able to engage the medial part of the sinus wall for apical support for the implant [17]. The relation between primary stability and the conical shape and design of this type of implant had also been pointed out earlier by Norton [25].

### 3.6. Bone Formation

After the sinus-lift surgery as described above, there are several local factors that may be important to the anticipated bone formation. Anatomical, prosthetic, surgical/technical, and patient-related variations and difficulties have to be evaluated in every case and may influence the outcome.

The newly formed bone around an implant installed with this technique is repeatedly seen on panoramic radiographs and resembles the bone seen around natural teeth in the maxillary sinus region. The first histological evidence to describe this special bone formation was published in 2006 by Palma et al. [26], where blood alone or autogenous bone graft in a sinus lift study in four primates were compared. Both test and control sides revealed no differences in bone formation, but the importance of the implant surface characteristics became evident as well as the bone forming capacity of the Schneiderian mucous membrane. More bone was formed on the oxidized modified surface than the control turned surface. In a similar way, more bone was also observed forming in the nonaugmented sides with blood only, along the top of the implants where the sinus mucosa was resting. One very important point may be that the grafted autogenous bone had to be replaced before new bone formation could occur in comparison with direct formation of bone from the blood clot. This event perhaps gives rise to “blocking” of the bone formation by the inflammation and removal that needs to take place in replacing old bone with new.

Recently, Kim et al. used a dog model to study the bone formation around implants under the sinus membrane protruding 8 mm into the maxillary sinus. The authors found extensive collapse of the clot and membrane resulting in rather minimal formation of new bone. They recommended that this method be used in cases when only a small amount of new bone was needed around implants placed simultaneously in the maxillary sinus floor [27].

The tenting of the sinus mucous membrane by the implants in the sinus floor is, of course, important for the clot formation and subsequent bone formation. The tissue formed by the clot under the elevated membrane is an unstable stage in the bone formation process, as also discussed by Xu et al. 2005 [28]. In their rabbit study, the sinus membranes were elevated and a clot was allowed to form; the newly formed clot decreased in volume significantly during the first weeks of healing, indicating the importance of a space holder such as an implant or other device. Sul et al. [29] evaluated different lengths of installed implants into the sinus cavity. They could see no difference on bone formation using 4 and 8 mm implants.

These studies question the bone forming capability of the technique. The new bone formed with this technique is seen in the marginal part around the implants. This resembles the situation in the human anatomy of the sinus floor with protruding roots of the teeth, often covered only by a thin layer of bone.

Cricchio et al. explored this problem in placing a resorbable space-making device in the sinus floor in six primates for a two-stage procedure, aiming at later implants installation. Even though the device had shortcomings regarding stability, the device was found histologically not to trigger any inflammation and succeeded in enabling formation of bone seen after 6 months [30]. Johansson et al. recently reported on the use of a hollow hydroxyapatite space-maintaining device in three patients for preventing the clot collapsing and enabling bone regeneration and subsequent implant installation [31].

Lundgren suggests that the sinus membrane should be sutured to the superior part of the bony window after elevation to prevent collapse of the membrane and to enable stable clot formation [14]. Other workers did not perform this manipulation in their studies, and the question remains to be solved whether this is significantly important or not [1, 24, 32].

There is also a difference in technique between studies, as some remove and replace the bony window and some keep it attached to the sinus mucosa elevating it up- and inwards into the maxillary sinus. In the study by Sohn et al. the bony window was replaced on one side by a resorbable membrane in 5 patients. In the other 5 patients, the bony window was used to seal the lateral wall of the sinus. No differences in outcome were reported. The technique using the bony window was shown to take less time and was also a less expensive solution for sealing the lateral wall [19].

Srouji and coauthors recently attempted to explain the formation of bone beneath the sinus membrane on the maxillary sinus floor by exploring the osteogenic potential of the Schneiderian maxillary sinus membrane as an explanation for the clinically observed induction of bone formation. In their first paper from 2009, human samples of the membrane were cultured and studied histologically [33]. Flow cytometry analysis proved the cells capable of inducing and expressing different osteogenic markers including alkaline phosphatase, bone morphogenic protein-2, osteopontin, osteonectin, and osteocalcin and of further mineralizing their extracellular matrix. Cultured cells and a ceramic mix (HA/*β*-TCP) were combined into a fibrin clot and subcutaneously implanted in a thymic nude mice. Bone of human origin was seen being formed over the surface of the carrier particles after 8 weeks of healing. The paper left the remaining question of where exactly in the cellular compartments of the Schneiderian membrane the osteogenic progenitor cells were located. The deeper layers of the membrane, with periosteum-like structure, and microvascular cells within the membrane may both serve as sources for the osteogenic capacity of the membrane and subsequent bone formation. In the second paper, human Schneiderian membrane was folded around a fibrin clot, which was then transplanted into mice [34]. As a result, ectopic bone formation was seen in the pocket. Dispase digestion was used to eliminate the epithelial layer, leaving the lamina propria to be transplanted subcutaneously with the periosteal layers facing each other. The scaffold, in this case a fibrin clot, was shown to be important for bone formation together with the osteogenic cells, as the absence of a fibrin clot resulted in significantly less formation (as did fibrin only as control with minimal or no formation of new ectopic bone).

The technique described requires a more invasive surgical approach than the transalveolar osteotome technique originally presented by Summers [35, 36]. In the paper by Jensen and Terheyden, 16 studies of transalveolar sinus floor elevation were identified. Of these studies, three reported data on the technique performed without the introduction of grafting material [37–39] using only blood around the implants elevating the sinus mucosa. After up to 25 months of loading, the median survival rate was 96% (186 implants in 110 patients). It could be argued that the transalveolar technique would be the method of choice due to its relative simplicity and low morbidity. On the other hand, the technique with the lateral window approach offers the possibility of controlling the sinus membrane and allows a wide dissection of the sinus membrane, hence minimizing the risk of sinus membrane perforations. Long implants are also able to be installed with a good overview and control through the bony window, eventually resulting in higher bone formation along these implants [17]. Additionally, the lateral window technique combined with use of a favourable implant design offers the possibility of treating cases with bone levels as low as 1-2 millimetres [16, 17]. Complete edentulous cases may therefore be treated with multiple sinus implants for a fixed restoration without the use of previous grafting. No studies comparing these two techniques could be identified in the literature so far, neither experimental nor clinical.

### 3.7. Potential Difficulties and Complications

The membrane elevation technique without the use of grafting materials, as described in this paper, is not initially an easy technique as it may require adaptation from the more common technique using grafts and where the bony window is prepared with a burr [1, 40]. The bony window needs to be removed from the sinus membrane, and piezosurgery may be advantageous when performing this stage in the procedure. However, with results comparable with routine sinus lift techniques, the membrane elevation technique displays possible advantages. The problems encountered, such as sinus septae and membrane perforations, are still factors that need to be taken in consideration.

The maxillary sinus is often divided into compartments by complete, or incomplete, bony septae. These must be accounted for in the surgical planning of the procedure and are best visualised by preoperative CT scanning [41]. The premolar region is also the location of most septae in atrophic edentulous ridges, and it has been shown that septae in dentate maxillas are of greater height than in edentulous patients [42]. When planning surgery, these septae may not only be a problem during the procedure but may also be helpful in achieving satisfactory primary stability for the implant when placed in these septae of the basal bone of the maxillary atrophied crest. The use of a wide bony window for access to the sinus mucosa is important. These anatomical features, septae and a fragile mucosa, may develop into lacerations of the sinus mucosa during the dissection, which needs to be addressed to complete the procedure. Suturing of the mucosa to the superior part of the bony window after extensive dissection has been recommended but not yet evaluated in controlled studies [14, 17].

Jung et al. evaluated the significance of perforation of the Schneiderian membrane during implant installation in the sinus floor [43]. Implants were allowed to penetrate up into the maxillary sinuses of eight dogs. The implants were placed so that 2, 4, or 8 mm of the implant surface was uncovered by bone in the bottom of the sinus, as observed through the bone window and the intentionally made laceration of the membrane. The dogs were killed after 6 months of healing. No signs of sinus disorder were seen in the dogs, also verified with CT scans after six months.

Implants penetrating with 2 mm into the sinus showed overgrowth with a new membrane. This new covering membrane (called a functional barrier) was not seen in the 4 and 8 mm groups, but the membrane was there found, without inflammatory signs, more to the base of the well-osseointegrated implants with direct attachment to the titanium implant surface.

In a retrospective study on humans, Jung et al. reported a similar lack of complications as seen in dogs. Nine patients with 23 implants inserted in the maxillary sinus were evaluated for sinus complications 6–10 months after insertion. No clinical signs of sinusitis were found although CT scans showed postoperative mucous thickening around 14 of the 23 implants [44]. If a perforation occurs, it might not be devastating to the operation.

In a prospective study of 100 cases, Wallace et al. found that the complication of perforations during surgery could be significantly reduced with the piezotechnique compared to the use of rotating instruments. The authors also pointed out that the perforations occurred during the hand instrumentation phase and not during the use of piezosurgery performed osteotomies [45].

The time needed for adequate maturation of new bone prior to loading of implants placed in low initial bone height is not well understood and needs further study.

## 4. Conclusion

The technique presented offers a method of augmenting the posterior maxilla when remaining bone levels in the edentulous region are low. The technique is now recognized as reliable and established [23, 46, 47]. The innate osteogenic potential of the Schneiderian membrane may be a main reason for the successful formation of bone with this augmentation technique. It is cost-effective as it is graft-less, less time-consuming, and comparatively inexpensive. Morbidity is lower than autogenous bone grafting since no extra graft material is needed.

## Figures and Tables

**Table 1:** Summary of published studies (1997–2011) of sinus lift with blood only with a follow-up of one year or more.

Study	number of patients	number of implants	Implant type	One-stage/ two-stage	Implant surface	Followup (mo)	Baseline bonelevel (mm)	Bone gain (mm)	Perforation of sinus (%)	Survival (%)
Ellegaard et al. [1]	24	26	Astra	Two-stage	TiO2-blasted	29,9 (average)	≥3	“Bone gain in most of the implants”	“In a few cases away from the implant site”	95%
		12	ITI	Two-stage	Solid screw	25,3 (average)				86%
Lundgren et al. [14]	10	19	TiUnite	Two-stage	Anodic oxidized	12	7 (range 4–10)	“In all patients bone formation was seen”	Perforation is described but ND	100%
Ellegaard et al. [15]	68	59	Astra	Two-stage	TiO2-blasted	64.2 (0–128)	≥3	ND	ND	10 yrs 85.4%
		72	ITI	One-stage	Solid screw	57.5 (0–143)				10 yrs 79.9%
Chen et al. [16]	33	18	ITI and Swissplus	One-stage	ND	24	7.5 ± 2.1	4.5 (range 3–9)	0	100%
		29	Frialit-2	Two-stage	ND					
Thor et al. [17]	20	44	Astra	Two-stage	TiO2-blasted	14–45	range 2–9	6.51 (range 4–10)	41	97.7%
Hatano et al. [18]	6	14	TiUnite	Two-stage	Anodic oxidized	12–34	range 2–10	ND	0	92,9%
Sohn et al. [19]	10	21	Seven	Two-stage	Sand-blasted and acid-etched	8.5 (range 6–12)	5 (range 1–9)	“All cases revealed bone formation”	Perforation is described but ND	100%
Balleri et al. [20]	15	28	Astra	Two-stage	TiO2-blasted	12	6.2 (range 4–10)	5.5 (range 3–8.2)	3 cases unknown number of sinuses	100%
Lin et al. [21]	44	80	ITI, Swissplus and Frialit-2	Mixed	ND	60	5.1 (range 4.6–6.6)	7.4 (range 5.7–9.1)	ND	100%
Cricchio et al. [22]	84	179	TiUnite	Two-stage	Anodic oxidized	12–72	5,7 (range 3.4–8)	5,2 (range 3–7.4)	11	99%

ND: no data.
